# Supplementary material for: The Association Between Delivery of Small-for-Gestational-Age Neonate and Their Risk for Long-Term Neurological Morbidity
Source: J Clin Med. 2020 Oct 2;9(10):3199. doi: 10.3390/jcm9103199 (PMC7599795; doi:10.3390/jcm9103199)
Supplement: Supplementary file 1 [file jcm-09-03199-s001.pdf]

Supplementary Table S1. *Neurologic diagnoses according to the International Classification of Diseases (ICD-9)*
Diagnosis code Diagnosis description

Autism

2990 Autistic disorder
2990 Infantile autism
2998 Other specified pervasive developmental disorders
29900 Autistic disorder, current or active state
29901 Autistic disorder, residual state
29910 Childhood disintegrative disorder, current or active state
29981 Other specified pervasive developmental disorders, residual state
29990 Unspecified pervasive developmental disorder, current or active state

Eating disorders

3071 Anorexia nervosa
3075 Other and unspecified disorders of eating
30750 Eating disorder, unspecified
30751 Bulimia nervosa
30753 Rumination disorder
30759 Other disorders of eating
V691 Inappropriate diet and eating habits

Sleep disorders

32727 Central sleep apnea in conditions classified elsewhere
78051 Insomnia with sleep apnea
78051 Insomnia with sleep apnea, unspecified
3073 Stereotypic movement disorder
7805 Sleep disturbances
30746 Sleep arousal disorder
30746 Somnambulism or night terrors
30747 Other dysfunctions of sleep stages or arousal from sleep
32730 Circadian rhythm sleep disorder, unspecified
32732 Circadian rhythm sleep disorder, advanced sleep phase type
34700 Narcolepsy without cataplexy
34701 Narcolepsy with cataplexy
78050 Unspecified sleep disturbance
78052 Insomnia, unspecified
78052 Other insomnia
78054 Hypersomnia, unspecified
78056 Dysfunctions associated with sleep stages or arousal from sleep
78059 Other sleep disturbances
V694 Lack of adequate sleep

Movement disorders

3331 Essential and other specified forms of tremor
3332 Myoclonus
3335 Other choreas
3336 Genetic torsion dystonia
3336 Idiopathic torsion dystonia
3343 Other cerebellar ataxia
3450 Generalized nonconvulsive epilepsy
3452 Petit mal status, epileptic
3453 Grand mal status, epileptic

3455 Partial epilepsy, without impairment of consciousness
3456 Infantile spasms

- 3459 Epilepsy, unspecified
- 7810 Abnormal involuntary movements
- 7812 Abnormality of gait
- 7813 Lack of coordination
- 33390 Unspecified extrapyramidal disease ■ abnormal movement disorder
- 33399 Other extrapyramidal diseases and abnormal movement disorders
- 34500 Generalized nonconvulsive epilepsy without intractable epilepsy
- 34501 Generalized nonconvulsive epilepsy with intractable epilepsy
- 34510 Generalized convulsive epilepsy without intractable epilepsy
- 34511 Generalized convulsive epilepsy with intractable epilepsy
- 34540 Partial epilepsy ■ impairment of consciousness without intractable epilepsy
- 34550 Partial epilepsy without impairment of consciousness without intractable epilepsy
- 34560 Infantile spasms without intractable epilepsy
- 34590 Epilepsy, unspecified without intractable epilepsy
- 34590 Epilepsy, unspecified without intractable epilepsy
- 34591 Epilepsy unspecified with intractable epilepsy
- 78031 Febrile convulsions
- 78031 Febrile convulsions (simple), unspecified
- 78032 Complex febrile convulsions
- 78039 Other convulsions
- 78099 Other general symptoms

Cerebral palsy

- 3341 Hereditary spastic paraplegia
- 3421 Spastic hemiplegia
- 3429 Hemiplegia, unspecified
- 3430 Congenital diplegia
- 3431 Congenital hemiplegia
- 3432 Congenital quadriplegia
- 3439 Infantile cerebral palsy, unspecified
- 3441 Paraplegia
- 3442 Diplegia of upper limbs
- 3449 Paralysis, unspecified
- 3481 Anoxic brain damage
- 3526 Multiple cranial nerve palsies
- 7814 Transient paralysis of limb
- 34210 Spastic hemiplegia affecting unspecified side
- 34290 Hemiplegia, unspecified, affecting unspecified side
- 34291 Hemiplegia, unspecified, affecting dominant side
- 34292 Hemiplegia, unspecified, affecting nondominant side
- 34400 Quadriplegia, unspecified
- 34430 Monoplegia of lower limb, affecting unspecified side
- 34440 Monoplegia of upper limb, affecting unspecified side
- 34489 Other specified paralytic syndrome
- 43811 Aphasia

Psychiatric emotional

- 309 Adjustment reaction
- 311 Depressive disorder, not elsewhere classified
- 316 Psychic factors associated with diseases classified elsewhere
- 2930 Acute delirium
- 2930 Delirium due to conditions classified elsewhere

2940 Amnestic disorder in conditions classified elsewhere
2949 Unspecified persistent mental disorders due to conditions classified elsewhere
2971 Delusional disorder
2979 Unspecified paranoid state
2981 Excitative type psychosis
2983 Acute paranoid reaction
2989 Unspecified psychosis
3003 Obsessive–compulsive disorders
3004 Dysthymic disorder
3004 Neurotic depression
3009 Unspecified nonpsychotic mental disorder
3019 Unspecified personality disorder
3026 Disorders of psychosexual identity
3051 Tobacco use disorder (tobacco dependence

3061 Respiratory malfunction arising from mental factors
3062 Cardiovascular malfunction arising from mental factors
3068 Other specified psychophysiological malfunction
3069 Unspecified psychophysiological malfunction
3070 Adult onset fluency disorder
3070 Stammering and stuttering
3070 Stuttering
3080 Predominant disturbance of emotions
3089 Unspecified acute reaction to stress
3090 Adjustment disorder with depressed mood
3094 Adjustment disorder with mixed disturbance of emotions and conduct
3099 Unspecified adjustment reaction
3129 Unspecified disturbance of conduct
3139 Unspecified emotional disturbance of childhood or adolescence
7801 Hallucinations
7803 Convulsions
7992 Nervousness
7993 Debility, unspecified
29384 Anxiety disorder in conditions classified elsewhere
29530 Paranoid type schizophrenia, unspecified state

29570 Schizoaffective disorder schizophrenia, unspecified state
29580 Other specified types of schizophrenia, unspecified state
29590 Unspecified type schizophrenia, unspecified state
29600 Bipolar i disorder, single manic episode, unspecified degree
29620 Major depressive affective disorder, single episode, unspecified degree

29680 Bipolar disorder, unspecified
29690 Unspecified episodic mood disorder
29699 Other specified affective psychoses
30000 Anxiety state, unspecified
30001 Panic disorder without agoraphobia
30009 Other anxiety states
30010 Hysteria, unspecified
30011 Conversion disorder
30029 Other isolated or simple phobias
30183 Borderline personality
30183 Borderline personality disorder
30302 Acute alcoholic intoxication in alcoholism, episodic drinking behavior

30400 Opioid type dependence, unspecified use
30430 Cannabis dependence, unspecified use
30432 Cannabis dependence, episodic use
30500 Alcohol abuse, unspecified drinking behavior
30501 Alcohol abuse, continuous drinking behavior
30502 Alcohol abuse, episodic drinking behavior
30591 Other, mixed, or unspecified drug abuse, continuous use
30720 Tic disorder, unspecified
30722 Chronic motor or vocal tic disorder
30723 Tourette's disorder
30752 Pica
30924 Adjustment disorder with anxiety
30981 Posttraumatic stress disorder
31210 Undersocialized conduct disorder, unaggressive type, unspecified
31239 Other disorders of impulse control
31389 Other emotional disturbances of childhood or adolescence
79921 Nervousness
79922 Irritability
79925 Demoralization and apathy
79929 Other signs and symptoms involving emotional state
V6284 Suicidal ideation

Attention deficit/hyperactivity disorder

3142 Hyperkinetic conduct disorder of childhood
3149 Unspecified hyperkinetic syndrome of childhood
31400 Attention deficit disorder without hyperactivity
31401 Attention deficit disorder with hyperactivity
V400 Mental and behavioral problems with learning
V409 Unspecified mental or behavioral problem

Developmental disorders

317 Mild intellectual disabilities
317 Mild mental retardation
319 Unspecified intellectual disabilities
319 Unspecified mental retardation
3152 Other specific developmental learning difficulties
3154 Developmental coordination disorder
3158 Other specified delays in development
3159 Unspecified delay in development
7834 Lack of expected normal physiological development
7834 Lack of expected normal physiological development in childhood
31531 Expressive language disorder
31534 Speech and language developmental delay due to hearing loss
31539 Other developmental speech disorder
33183 Mild cognitive impairment, so stated
78340 Lack of normal physiological development, unspecified

Degenerative, demyelization

330 Cerebral degenerations usually manifest in childhood
335 Anterior horn cell disease
340 Multiple sclerosis

3300 Leukodystrophy
3308 Other specified cerebral degenerations in childhood
3313 Communicating hydrocephalus
3314 Obstructive hydrocephalus
3319 Cerebral degeneration, unspecified
3348 Other spinocerebellar diseases
3350 Werdnig–Hoffmann disease
3360 Syringomyelia and syringobulbia
3410 Neuromyelitis optica
3411 Schilder's disease
3419 Demyelinating disease of central nervous system, unspecified
3480 Cerebral cysts
3590 Congenital hereditary muscular dystrophy
3591 Hereditary progressive muscular dystrophy
33189 Other cerebral degeneration
33510 Spinal muscular atrophy, unspecified
33522 Progressive bulbar palsy
33523 Pseudobulbar palsy
34120 Acute (transverse) myelitis NOS
348891 Cerebral calcification
3313 2 Post hemorrhagic hydrocephalus

Myopathy

352 Disorders of other cranial nerves
3379 Unspecified disorder of autonomic nervous system
3510 Bell's palsy
3518 Other facial nerve disorders
3519 Facial nerve disorder, unspecified
3539 Unspecified nerve root and plexus disorder
3542 Lesion of ulnar nerve
3548 Other mononeuritis of upper limb
3549 Mononeuritis of upper limb, unspecified
3553 Lesion of lateral popliteal nerve
3556 Lesion of plantar nerve
3558 Mononeuritis of lower limb, unspecified
3559 Mononeuritis of unspecified site
3562 Hereditary sensory neuropathy
3564 Idiopathic progressive polyneuropathy
3568 Other specified idiopathic peripheral neuropathy
3569 Unspecified idiopathic peripheral neuropathy
3570 Acute infective polyneuritis
3571 Polyneuropathy in collagen vascular disease
3572 Polyneuropathy in diabetes
3577 Polyneuropathy due to other toxic agents
3588 Other specified myoneural disorders
3589 Myoneural disorders, unspecified
3592 Myotonic disorders
3599 Myopathy, unspecified
33709 Other idiopathic peripheral autonomic neuropathy
33720 Reflex sympathetic dystrophy, unspecified
33721 Reflex sympathetic dystrophy of upper limb
33722 Reflex sympathetic dystrophy of lower limb

35781 Chronic inflammatory demyelinating polyneuritis
35800 Myasthenia gravis without (acute) exacerbation
